# Poly[diaqua­(μ_3_-8-oxidoquinoline-5-sulfonato-κ^4^ *N*,*O* ^8^:*O* ^5^:*O* ^8^)nickel(II)]

**DOI:** 10.1107/S1600536809026105

**Published:** 2009-07-11

**Authors:** Ying Wang, Li Wang, Jianing Xu, Guangshan Zhu

**Affiliations:** a Key Laboratory of Inorganic Synthesis and Preparative Chemistry, Jilin University, Changchun 130012, People’s Republic of China

## Abstract

In title compound, [Ni(C_9_H_5_NO_4_S)(H_2_O)_2_]_*n*_, the Ni^II^ atom is coordinated by one N atom and two bridging O atoms from two 8-oxidoquinoline-5-sulfonate ligands, one sulfonate O atom from a third ligand, and two water mol­ecules in a distorted octa­hedral geometry. The two Ni^II^ atoms are linked to each other through the bridging O atoms, forming a dimer. Adjacent dimers are connected through the coordination of the sulfonate O atom into a two-dimensional coordination network parallel to (010). Hydrogen bonds between the coordinated water mol­ecules and the uncoordinated O atoms of the sulfonate groups result in the construction of a three-dimensional supra­molecular structure.

## Related literature

For related structures, see: Ammor *et al.* (1992 ▶); Petit *et al.* (1993*a* ▶,*b* ▶); Rao *et al.* (2003 ▶); Wu *et al.* (2008 ▶); Xie *et al.* (1992 ▶).
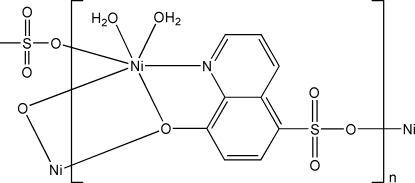


## Experimental

### 

#### Crystal data

[Ni(C_9_H_5_NO_4_S)(H_2_O)_2_]
*M* *_r_* = 317.94
Orthorhombic, 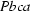

*a* = 9.2067 (8) Å
*b* = 15.0504 (13) Å
*c* = 16.1599 (14) Å
*V* = 2239.2 (3) Å^3^
*Z* = 8
Mo *K*α radiation
μ = 1.94 mm^−1^
*T* = 293 K
0.28 × 0.22 × 0.18 mm

#### Data collection

Bruker SMART APEX CCD diffractometer
Absorption correction: multi-scan (*SADABS*; Bruker, 2001 ▶) *T*_min_ = 0.601, *T*_max_ = 0.701
11973 measured reflections
2198 independent reflections
1874 reflections with *I* > 2σ(*I*)
*R*_int_ = 0.037

#### Refinement

*R*[*F* ^2^ > 2σ(*F* ^2^)] = 0.031
*wR*(*F* ^2^) = 0.079
*S* = 1.02
2198 reflections
171 parameters
4 restraints
H atoms treated by a mixture of independent and constrained refinement
Δρ_max_ = 0.64 e Å^−3^
Δρ_min_ = −0.27 e Å^−3^

### 

Data collection: *SMART* (Bruker, 2007 ▶); cell refinement: *SAINT* (Bruker, 2007 ▶); data reduction: *SAINT*; program(s) used to solve structure: *SHELXS97* (Sheldrick, 2008 ▶); program(s) used to refine structure: *SHELXL97* (Sheldrick, 2008 ▶); molecular graphics: *SHELXTL* (Sheldrick, 2008 ▶) and *DIAMOND* (Brandenburg, 1999 ▶); software used to prepare material for publication: *SHELXTL*.

## Supplementary Material

Crystal structure: contains datablocks global, I. DOI: 10.1107/S1600536809026105/hy2203sup1.cif

Structure factors: contains datablocks I. DOI: 10.1107/S1600536809026105/hy2203Isup2.hkl

Additional supplementary materials:  crystallographic information; 3D view; checkCIF report

## Figures and Tables

**Table 1:** Selected bond lengths (Å)

Ni1—O1^i^	2.0153 (17)
Ni1—O6*W*	2.0285 (19)
Ni1—O1	2.0443 (16)
Ni1—N1	2.052 (2)
Ni1—O5*W*	2.0936 (19)
Ni1—O2^ii^	2.1437 (17)

Symmetry codes: (i) 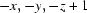; (ii) 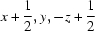.

**Table 2:** Hydrogen-bond geometry (Å, °)

*D*—H⋯*A*	*D*—H	H⋯*A*	*D*⋯*A*	*D*—H⋯*A*
O5*W*—H5*WA*⋯O3^iii^	0.82	2.00	2.812 (2)	171
O5*W*—H5*WB*⋯O4^iv^	0.80 (2)	2.07 (2)	2.866 (3)	170 (2)
O6*W*—H6*WA*⋯O3^iv^	0.82	1.93	2.687 (2)	153
O6*W*—H6*WB*⋯O4^v^	0.78 (2)	2.04 (2)	2.787 (3)	159 (3)

Symmetry codes: (iii) 
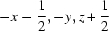
; (iv) 
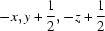
; (v) 
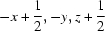
.

**Fig. 1.**
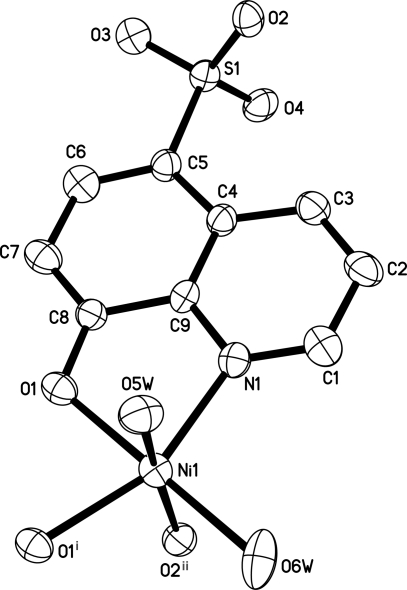
Coordination environment of the Ni atom in the title compound. Displacement ellipsoids are drawn at the 50% probability level. [Symmetry code: (i) -x,-y,-z + 1; (ii) x + 1/2, y, -z + 1/2.]

**Fig. 2.**
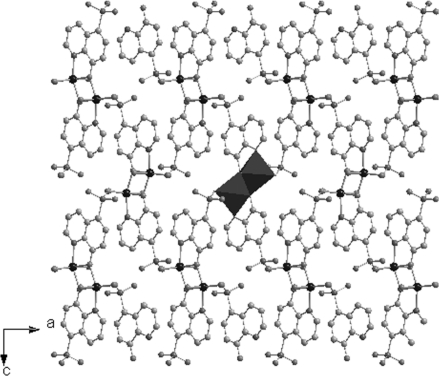
The two-dimensional coordination network of the title compound with a (4,4) topology, viewed along the [0 1 0] direction.

**Fig. 3.**
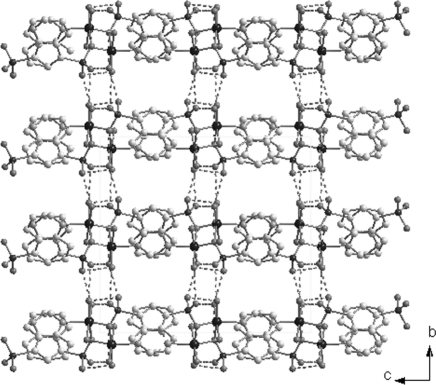
The three-dimensional supramolecular structure of the title compound, connected by hydrogen bonds between the coordinated water molecules and the uncoordinated sulfonate O atoms.

## supplementary crystallographic information

### Comment

The metal complexes with organic ligands containing sulfonate group still remain largely unexplored. We have been investigating the formation of novel transition metal coordination polymers employing hydrothermal methods. During the course of the investigation employing 8-hydroxylquinoline-5-sulfonic acids (H_2_QS) as organic ligand, which has received a little attention (Ammor *et al.*, 1992; Petit *et al.*, 1993*a*,b; Rao *et al.*, 2003; Wu *et al.*, 2008; Xie *et al.*, 1992), and nickel(II) as metal center, we isolated a new two-dimensional coordination polymer.

As shown in Fig. 1, the asymmetric unit of the title compound contains one Ni^II^ atom, one QS ligand, and two water molecules. The Ni^II^ atom adopts a distorted octahedral coordination geometry, defined by one N atom and two bridging olate O atoms from two QS ligands, one sulfonate O atom from a third ligand, and two water molecules (Table 1). Two crystallographically equivalent Ni atoms [Ni1 and Ni1^i^, symmetry code: (i) -*x*, -*y*, 1 - *z*] link to each other through two bridging atoms O1 and O1^i^, forming an edge-sharing dimer. These dimers are connected by the sulfonate groups of the QS ligands into an infinite two-dimensional coordination network with a (4,4) topology along the [0 1 0] direction, as shown in Fig. 2. These networks are further connected by hydrogen bonds between the coordinated water molecules and the uncoordinated O atoms of the sulfonate groups into a three-dimensional supramolecular structure (Fig. 3 and Table 2).

### Experimental

A mixture of Ni(NO_3_)_2_.4H_2_O (0.250 g, 1 mmol) and H_2_QS (0.024 g, 0.1 mmol) was dissolved in 6 ml H_2_O with stirring about half an hour and pH = 6. The mixture was transferred to a 15 ml Teflon-lined stainless-steel hydrothermal autoclave and heated at 413 K for two weeks under autogenous pressure. The green block crystals were filtered off, washed with ethanol and dried at room temperature.

### Refinement

H atoms on C atoms are positioned geometrically and refined as riding atoms, with C—H = 0.93 Å and *U*_iso_ = 1.2*U*_eq_(C). H atoms of water molecules are located in a difference Fourier map. Two H atoms (H5WA and H6WA) were refined as riding atoms, with O—H = 0.82 Å and *U*_iso_ = 1.5*U*_eq_(O), and the other two (H5WB and H6WB) were refined isotropically.

### Crystal data

**Table:**

[Ni(C_9_H_5_NO_4_S)(H_2_O)_2_]	*F*(000) = 1296
*M**_r_* = 317.94	*D*_x_ = 1.886 Mg m^−^^3^*D*_m_ = 1.886 Mg m^−^^3^*D*_m_ measured by not measured
Orthorhombic, *P**b**c**a*	Mo *K*α radiation, λ = 0.71073 Å
Hall symbol: -P 2ac 2ab	Cell parameters from 2198 reflections
*a* = 9.2067 (8) Å	θ = 2.5–28.1°
*b* = 15.0504 (13) Å	µ = 1.94 mm^−^^1^
*c* = 16.1599 (14) Å	*T* = 293 K
*V* = 2239.2 (3) Å^3^	Block, green
*Z* = 8	0.28 × 0.22 × 0.18 mm

### Data collection

**Table:**

Bruker SMART APEX CCD diffractometer	2198 independent reflections
Radiation source: fine-focus sealed tube	1874 reflections with *I* > 2σ(*I*)
graphite	*R*_int_ = 0.037
φ and ω scans	θ_max_ = 26.0°, θ_min_ = 2.5°
Absorption correction: multi-scan (*SADABS*; Bruker, 2001)	*h* = −10→11
*T*_min_ = 0.601, *T*_max_ = 0.701	*k* = −18→17
11973 measured reflections	*l* = −18→19

### Refinement

**Table:**

Refinement on *F*^2^	Primary atom site location: structure-invariant direct methods
Least-squares matrix: full	Secondary atom site location: difference Fourier map
*R*[*F*^2^ > 2σ(*F*^2^)] = 0.031	Hydrogen site location: inferred from neighbouring sites
*wR*(*F*^2^) = 0.079	H atoms treated by a mixture of independent and constrained refinement
*S* = 1.02	*w* = 1/[σ^2^(*F*_o_^2^) + (0.0484*P*)^2^] where *P* = (*F*_o_^2^ + 2*F*_c_^2^)/3
2198 reflections	(Δ/σ)_max_ = 0.001
171 parameters	Δρ_max_ = 0.64 e Å^−^^3^
4 restraints	Δρ_min_ = −0.27 e Å^−^^3^

### Fractional atomic coordinates and isotropic or equivalent isotropic displacement parameters (Å^2^)

**Table:**

	*x*	*y*	*z*	*U*_iso_*/*U*_eq_	
Ni1	0.10943 (3)	0.058524 (19)	0.446648 (17)	0.02363 (12)	
O5W	−0.0374 (2)	0.16474 (12)	0.44545 (10)	0.0326 (4)	
H5WA	−0.0848	0.1645	0.4884	0.049*	
O1	0.04736 (19)	0.03393 (11)	0.56593 (9)	0.0264 (4)	
N1	0.0998 (2)	0.05336 (12)	0.31986 (12)	0.0250 (5)	
C8	−0.0750 (3)	−0.05668 (14)	0.35675 (14)	0.0232 (5)	
C9	0.0015 (3)	−0.00829 (15)	0.29298 (13)	0.0231 (5)	
C4	−0.0248 (3)	−0.02414 (16)	0.20803 (13)	0.0251 (5)	
C2	0.1498 (3)	0.09088 (18)	0.17944 (15)	0.0338 (6)	
H2A	0.2007	0.1263	0.1423	0.041*	
C3	0.0542 (3)	0.02903 (17)	0.15126 (14)	0.0300 (6)	
H3B	0.0406	0.0216	0.0947	0.036*	
C5	−0.1275 (3)	−0.09119 (16)	0.18792 (14)	0.0256 (5)	
C1	0.1711 (3)	0.10083 (16)	0.26430 (15)	0.0311 (6)	
H1B	0.2383	0.1426	0.2826	0.037*	
C6	−0.1969 (3)	−0.13796 (16)	0.24900 (15)	0.0310 (6)	
H6A	−0.2631	−0.1819	0.2342	0.037*	
C7	−0.1711 (3)	−0.12159 (15)	0.33312 (15)	0.0314 (6)	
H7A	−0.2193	−0.1549	0.3731	0.038*	
O6W	0.2752 (2)	0.14637 (13)	0.45841 (13)	0.0415 (5)	
H6WA	0.2501	0.1948	0.4401	0.062*	
H5WB	−0.011 (3)	0.2155 (12)	0.4428 (14)	0.029 (7)*	
H6WB	0.347 (3)	0.134 (2)	0.4814 (19)	0.060 (11)*	
S1	−0.16782 (7)	−0.11580 (4)	0.08365 (4)	0.02460 (16)	
O2	−0.21464 (19)	−0.03403 (11)	0.04239 (9)	0.0293 (4)	
O4	−0.03579 (19)	−0.15043 (12)	0.04607 (10)	0.0352 (4)	
O3	−0.28431 (19)	−0.18166 (11)	0.08585 (10)	0.0313 (4)	

### Atomic displacement parameters (Å^2^)

**Table:**

	*U*^11^	*U*^22^	*U*^33^	*U*^12^	*U*^13^	*U*^23^
Ni1	0.0259 (2)	0.0217 (2)	0.02325 (19)	−0.00303 (12)	−0.00136 (12)	0.00040 (11)
O5W	0.0340 (11)	0.0262 (10)	0.0375 (10)	0.0004 (8)	0.0078 (8)	0.0026 (8)
O1	0.0293 (10)	0.0275 (9)	0.0225 (8)	−0.0058 (8)	0.0005 (7)	−0.0008 (7)
N1	0.0235 (11)	0.0235 (11)	0.0279 (11)	−0.0013 (8)	−0.0027 (8)	−0.0001 (8)
C8	0.0250 (13)	0.0217 (12)	0.0230 (12)	0.0009 (9)	0.0008 (9)	0.0002 (9)
C9	0.0191 (11)	0.0232 (12)	0.0270 (12)	0.0017 (9)	−0.0022 (9)	−0.0010 (9)
C4	0.0246 (13)	0.0263 (12)	0.0245 (12)	0.0038 (10)	−0.0011 (10)	−0.0017 (9)
C2	0.0348 (15)	0.0385 (15)	0.0282 (13)	−0.0071 (12)	0.0025 (11)	0.0077 (12)
C3	0.0305 (14)	0.0348 (14)	0.0246 (12)	−0.0007 (11)	−0.0002 (10)	0.0009 (10)
C5	0.0259 (13)	0.0259 (12)	0.0250 (12)	0.0028 (10)	−0.0012 (10)	−0.0020 (10)
C1	0.0317 (14)	0.0299 (14)	0.0317 (13)	−0.0106 (11)	−0.0051 (11)	0.0019 (10)
C6	0.0338 (14)	0.0265 (13)	0.0327 (13)	−0.0065 (11)	−0.0032 (11)	−0.0038 (11)
C7	0.0358 (15)	0.0308 (14)	0.0276 (13)	−0.0072 (11)	0.0013 (11)	0.0017 (10)
O6W	0.0318 (11)	0.0256 (10)	0.0670 (13)	−0.0053 (8)	−0.0201 (10)	0.0100 (9)
S1	0.0260 (3)	0.0232 (3)	0.0246 (3)	0.0021 (2)	−0.0014 (2)	−0.0041 (2)
O2	0.0330 (10)	0.0263 (9)	0.0284 (9)	0.0054 (8)	−0.0022 (7)	0.0002 (7)
O4	0.0320 (10)	0.0358 (11)	0.0377 (10)	0.0081 (8)	0.0043 (8)	−0.0047 (8)
O3	0.0353 (10)	0.0276 (9)	0.0310 (9)	−0.0038 (8)	−0.0036 (7)	−0.0052 (7)

### Geometric parameters (Å, °)

**Table:**

Ni1—O1^i^	2.0153 (17)	C4—C5	1.421 (3)
Ni1—O6W	2.0285 (19)	C2—C3	1.360 (4)
Ni1—O1	2.0443 (16)	C2—C1	1.393 (3)
Ni1—N1	2.052 (2)	C2—H2A	0.9300
Ni1—O5W	2.0936 (19)	C3—H3B	0.9300
Ni1—O2^ii^	2.1437 (17)	C5—C6	1.371 (3)
O5W—H5WA	0.8200	C5—S1	1.765 (2)
O5W—H5WB	0.804 (17)	C1—H1B	0.9300
O1—C8^i^	1.320 (3)	C6—C7	1.402 (3)
N1—C1	1.322 (3)	C6—H6A	0.9300
N1—C9	1.367 (3)	C7—H7A	0.9300
C8—O1^i^	1.320 (3)	O6W—H6WA	0.8200
C8—C7	1.372 (3)	O6W—H6WB	0.784 (17)
C8—C9	1.445 (3)	S1—O4	1.4553 (18)
C9—C4	1.414 (3)	S1—O3	1.4608 (17)
C4—C3	1.418 (3)	S1—O2	1.4645 (17)
			
O1^i^—Ni1—O6W	176.93 (8)	C9—C4—C5	117.1 (2)
O1^i^—Ni1—O1	76.69 (7)	C3—C4—C5	126.5 (2)
O6W—Ni1—O1	103.89 (8)	C3—C2—C1	119.6 (2)
O1^i^—Ni1—N1	80.92 (7)	C3—C2—H2A	120.2
O6W—Ni1—N1	98.67 (8)	C1—C2—H2A	120.2
O1—Ni1—N1	157.28 (7)	C2—C3—C4	120.1 (2)
O1^i^—Ni1—O5W	93.64 (8)	C2—C3—H3B	119.9
O6W—Ni1—O5W	89.40 (8)	C4—C3—H3B	119.9
O1—Ni1—O5W	88.06 (7)	C6—C5—C4	120.7 (2)
N1—Ni1—O5W	89.54 (7)	C6—C5—S1	118.81 (19)
O1^i^—Ni1—O2	59.09 (4)	C4—C5—S1	120.49 (18)
O6W—Ni1—O2	120.98 (6)	N1—C1—C2	122.7 (2)
O1—Ni1—O2	133.91 (5)	N1—C1—H1B	118.6
N1—Ni1—O2^ii^	95.19 (7)	C2—C1—H1B	118.6
O5W—Ni1—O2^ii^	170.00 (7)	C5—C6—C7	121.9 (2)
Ni1—O5W—H5WA	109.5	C5—C6—H6A	119.0
Ni1—O5W—H5WB	122 (2)	C7—C6—H6A	119.0
H5WA—O5W—H5WB	102.2	C8—C7—C6	120.3 (2)
C8^i^—O1—Ni1^i^	114.35 (14)	C8—C7—H7A	119.9
C8^i^—O1—Ni1	142.34 (15)	C6—C7—H7A	119.9
Ni1^i^—O1—Ni1	103.31 (7)	Ni1—O6W—H6WA	109.5
C1—N1—C9	118.7 (2)	Ni1—O6W—H6WB	122 (2)
C1—N1—Ni1	129.50 (16)	H6WA—O6W—H6WB	128.7
C9—N1—Ni1	111.81 (15)	O4—S1—O3	112.36 (11)
O1^i^—C8—C7	124.9 (2)	O4—S1—O2	110.91 (10)
O1^i^—C8—C9	116.8 (2)	O3—S1—O2	111.42 (10)
C7—C8—C9	118.3 (2)	O4—S1—C5	107.33 (11)
N1—C9—C4	122.4 (2)	O3—S1—C5	105.87 (10)
N1—C9—C8	115.98 (19)	O2—S1—C5	108.68 (10)
C4—C9—C8	121.6 (2)	S1—O2—Ni1^iii^	136.95 (11)
C9—C4—C3	116.4 (2)		

Symmetry codes: (i) −*x*, −*y*, −*z*+1; (ii) *x*+1/2, *y*, −*z*+1/2; (iii) *x*−1/2, *y*, −*z*+1/2.

### Hydrogen-bond geometry (Å, °)

**Table:**

*D*—H···*A*	*D*—H	H···*A*	*D*···*A*	*D*—H···*A*
O5W—H5WA···O3^iv^	0.82	2.00	2.812 (2)	171
O5W—H5WB···O4^v^	0.80 (2)	2.07 (2)	2.866 (3)	170 (2)
O6W—H6WA···O3^v^	0.82	1.93	2.687 (2)	153
O6W—H6WB···O4^vi^	0.78 (2)	2.04 (2)	2.787 (3)	159 (3)

Symmetry codes: (iv) −*x*−1/2, −*y*, *z*+1/2; (v) −*x*, *y*+1/2, −*z*+1/2; (vi) −*x*+1/2, −*y*, *z*+1/2.
